# Fostering Emotional Availability in Mother-Child-Dyads With an Immigrant Background: A Randomized-Controlled-Trial on the Effects of the Early Prevention Program First Steps

**DOI:** 10.3389/fpsyg.2022.790244

**Published:** 2022-04-07

**Authors:** Judith Lebiger-Vogel, Constanze Rickmeyer, Marianne Leuzinger-Bohleber, Patrick Meurs

**Affiliations:** ^1^Sigmund-Freud-Institut, Frankfurt, Germany; ^2^University Medical Center, Johannes Gutenberg University Mainz, Mainz, Germany; ^3^University of Kassel, Kassel, Germany; ^4^Katholieke Universiteit Leuven, Leuven, Belgium

**Keywords:** Emotional Availability Scales, mother-child relationship, migration, immigrant background, early prevention, psychoanalysis

## Abstract

**Background:**

In many Western countries like Germany, the social integration of children with an immigrant background has become an urgent social tasks. The probability of them living in high-risk environments and being disadvantaged regarding health and education-related variables is still relatively higher. Yet, promoting language acquisition is not the only relevant factor for their social integration, but also the support of earlier developmental processes associated with adequate early parenting in their first months of life. The Emotional Availability Scales (EAS) measure the quality of caregiver-child-interactions as an indicator of the quality of their relationship and thus of such early parenting, focusing on mutual and emotional aspects of their interaction.

**Method:**

This pilot study examined in a randomized controlled trial the effects of the prevention project First Steps regarding the hypothesis that the Emotional Availability (EA) improved to a greater extent in “difficult-to-reach” immigrant mother-child dyads in a psychoanalytically oriented early intervention (A, FIRST STEPS) compared to a usual care intervention (B) offered by paraprofessionals with an immigrant background. A sample of *N* = 118 immigrant women in Germany from 37 different countries and their children was compared with regard to the parental EA-dimensions *sensitivity*, *structuring*, *non-intrusiveness* and *non-hostility* and the child dimensions *responsiveness* to and *involvement* of the caregiver in the pre-post RCT design.

**Results and Conclusion:**

Different from what was expected, repeated ANOVAs revealed no significant pre-post group differences for the parental dimensions. For the child dimensions the effect of time of measurement was highly significant, which can be interpreted as mostly natural developmental effects. Still, on the level of simple main effects for each intervention, only in the FIRST STEPS groups child *responsiveness* significantly improved. When controlled for confounding variables, a significant interaction effect for maternal *sensitivity* in favor of the FIRST STEPS intervention was found. The systematic group differences indicate that the more extensive and professional intervention, focusing on the individual needs of the participants, is more suitable to support the quality of the mother-child-relationship amongst immigrant mother-child dyads than usual care. The results are discussed taking into account the context of the maternal migration process and potential maternal traumatization.

**Clinical Trial Registration:**

[https://clinicaltrials.gov], identifier [DRKS00004632].

## Introduction

In Germany, children with an immigrant background still have to face disadvantages regarding variables that are health related such as psychopathological problems or obesity (Kurth and Schaffrath Rosario, 2007; Hölling et al., 2008; Rattay et al., 2012), their educational success and the probability for them to live in high-risk environments is higher [Leuzinger-Bohleber et al., 2011; Autorengruppe Bildungsberichterstattung, 2020; Bundesministerium für Arbeit und Soziales [BMAS], 2021; Sachverständigenrat deutscher Stiftungen für Integration und Migration [SVR], 2021]. However, it is not the migration background *per se* that poses the risk of these children being disadvantaged, but rather factors associated with the migration of the parents, psychological factors in the context of different phases of the migration process and socio-economic factors (low socio-economic status, unemployment, insecure residence status, etc.), which create a difficult development environment for these children (Lebiger-Vogel et al., 2015; Merry et al., 2020).

Particularly disadvantaged are children of mothers who have just recently migrated to Germany, because the mothers themselves are in an emotionally insecure situation. They have to find their way in a new environment without experienced caregivers—such as their parents and siblings—available. Especially in the vulnerable time after the birth of a child (Merry et al., 2020), they often feel isolated and alone and the risks of social withdrawal, loneliness and depression are significant (Moro, 2014). These stresses and strains on the mothers can have a negative effect on the emotional quality of the early mother-child relationship and the security of attachment and thus bring additional disadvantages to the children, which assigns them to a special risk group of the immigrant population. This shapes the developmental context for the children and can have negative consequences for their development. A child who unconsciously perceives that its mother feels strange in the new country and is very homesick could get into a loyalty conflict in which integration into the country of immigration is experienced as a betrayal of the mother’s (parents) home country (King, 2007; Leuzinger-Bohleber and Lebiger-Vogel, 2016; Lebiger-Vogel et al., 2020). As a result, it can be quite ambivalent for a child to adopt a bicultural identity and to learn the language of the new country and its development can be further impaired if it gets stuck in these ambivalences (Rauwald, 2009, 2013). If parents have been unable to process their migration experiences sufficiently, the stressful effects of migration can be transmitted to the next generation, as Kogan (2005) described (see also Leuzinger-Bohleber and Hettich, 2018; Lebiger-Vogel et al., 2020). The grieving process that is necessary for a mature processing of the migration experience can be impaired by feelings of guilt toward those who remained in the country of origin, as well as previous traumatic experiences before or during the migration (Volkan, 2017). Especially for refugee families, it is important to reduce the likelihood of the parental trauma being transmitted on to the next generation (see Leuzinger-Bohleber and Lebiger-Vogel, 2016; Leuzinger-Bohleber and Hettich, 2018; Lebiger-Vogel et al., 2020). Furthermore, many are confronted with other stressful factors after their immigration (e.g., insecure residence status, social isolation, discrimination). A lack of early integration can be associated with a possible disruption of attachment development in the course of migration and young motherhood, as the results of the Frankfurt prevention study showed (Leuzinger-Bohleber et al., 2006, 2009, 2011).

In Germany there are a number of projects that promote the social integration of children with an immigrant background. The majority is aimed at older children or adults, as the focus is on learning the German language (Lösel et al., 2006; Friedrich and Siegert, 2009; Leuzinger-Bohleber, 2014) and the well-being of small children is not explicitly taken into account. Although in recent years more emphasis has been put on early child development (on infants and toddlers, from 0 to 3 years) and parenting skills (Bundesministerium für Familie, Senioren, Frauen und Jugend, 2020). This makes sense, because children do not start with their active language acquisition before their second year of life and during the first years of life, learning their mother’s tongue is most important. As is known, language development is based on earlier “embodied” experiences and preverbal relationships. Stern (1985) showed in his studies on the developmental stages of the self that the “verbal self” in the second year of life is based on earlier developmental stages (the emergent self, the core self, etc.). Empirical research results also suggest such an approach: Infants growing up in an emotionally secure and positive environment learn languages more easily, show less aggression, are more creative and show a better affective, cognitive, and socio-emotional development (van IJzendoorn et al., 1995; Aviezer et al., 2002; Sroufe et al., 2005; DeKlyen and Greenberg, 2008; Thompson, 2008; Fearon et al., 2010). It can therefore be assumed that approaches based on early relationships in the immediate living environment (at this age, especially the nuclear family) could improve the integration of children with a migration background in infancy and toddlerhood.

All these results and assumptions led to the development of the FIRST STEPS intervention, a psychoanalytically oriented prevention program for immigrant families, who mostly have a low socioeconomic status and are “difficult-to-reach”^1^; they generally use counseling services less frequently than many German families. The intervention was offered from birth of the child until them entering kindergarten. As described before, its focus is “on the specific challenges and needs of families with an immigrant background” seeking “to optimize the early developmental environment of children at risk of growing up disadvantaged due to their parents’ acute migration” (Lebiger-Vogel et al., 2015, p. 3; see also Leuzinger-Bohleber and Lebiger-Vogel, 2016; Lebiger-Vogel et al., 2020). The project has been inspired by the psychoanalytically oriented First Steps program by Meurs et al. (2006) in Belgium which started in 2000 and has already been evaluated. Their results indicate that children with an immigrant background and children with and without an immigrant background affected by poverty show developmental delays in different domains already during the first 3 years of life. In addition, Meurs et al. (2006) showed that their program helped to prevent early developmental delays in immigrant children, especially if they were affected by poverty, and that in the long term the program had positive effects on the school success of the children compared to a matched control group of children with an immigrant background (Meurs and Jullian, 2008, 2015; Lebiger-Vogel et al., 2020). Furthermore, to our knowledge, no other psychoanalytically oriented early prevention for immigrant children exists so far.

Emotional availability (EA), originally introduced by Mahler et al. (1975), is a construct describing an individual’s emotional responsiveness and “attunement” to another’s needs and goals (Emde, 1980). It refers to the ability of a dyad to establish an emotionally healthy connection including a wide range of emotions, positive as well as negative (Emde, 1980; Emde and Easterbrooks, 1985; Easterbrooks and Biringen, 2000). Bornstein et al. (2012) define “EA (…) [as] the open, eager, collaborative, reciprocal communication that can occur between a mother and infant under optimal conditions, regardless of their culture, place of residence, or socioeconomic status” (p. 114). EA, as described by Biringen (2008), is characterized by a dyadic perspective rather than a unidirectional reaction of the caregiver to the child’s signals, which means that both mother (caregiver) and child contribute to the overall quality of the interaction. The Emotional Availability Scales (EAS, Biringen, 2008) assess EA as a multidimensional construct, consisting of four dimensions focusing on the caregiver, and two dimensions focusing on the child. *Sensitivity* refers to adult qualities regarding the ability to be warm and emotionally connected with the child, closest connected to the classic concept by Ainsworth et al. (1974/1990, 1978). It comprises a positive, authentic and genuine affect as well as the congruence of verbal and non-verbal channels of (emotion) expression. *Structuring* refers to the extent to which the adult serves as a mentor guiding the child’s activities and providing a holding framework. *Non-intrusiveness* describes the absence of over-directive or over-stimulating behavior, unrequested interferences or over-protection of the caregiver. *Non-hostility* refers to the absence of hostile reactions of the caregiver, hidden or obvious, in deed or word (Biringen et al., 2014). *Responsiveness* of the child refers to the emotional as well as the social responsiveness of the child to the caregiver. It comprises both the affective and the behavioral quality of the child’s reaction, thus its eagerness, interest and pleasure to a parental invitation to interact (see also Rickmeyer et al., 2017). This scale reflects the concept that most closely approximates the current attachment view of a securely or insecurely attached child and relates to the child’s ability to explore independently of and respond affectively positive to the caregiver. Child’s *involvement* of the caregiver is about its ability to attend to interactions with its caregiver, to engage him or her and to invite the him/her into a playful exchange (see also Rickmeyer et al., 2017).

Overall, the EAS were applied in more than 22 countries, showing adequate validity and reliability in each of them [see review by Biringen et al. (2014)]. Positive associations of EA with attachment security have been reported for different samples from Europe, North-America, Japan and Israel (Easterbrooks and Biringen, 2000; Ziv et al., 2000; Biringen et al., 2008, 2012; Komatsu, 2011; Easterbrooks et al., 2012). Furthermore, studies reported positive associations with the child’s capacity for emotion regulation, sleep-wake-regulation or the social and language development [see review by Biringen et al. (2014)], parental knowledge about child-rearing in Italy, the United States and Argentina (Bornstein et al., 2008) and mothers psychosocial functioning in a Mexican group of immigrants in the United States (Howes and Hong, 2008). In addition, several studies have shown that samples at high risk of developmental problems due to various psychological or mental problems (e.g., history of abuse, substance abuse, maternal depression, etc.) have a comparatively lower EA [for an overview see Biringen et al. (2014); see also Rickmeyer et al. (2017)]. Only very few studies though, compared the cross-cultural level of EA among immigrants from different parts of the world but living in the same country or EA patterns in different countries. To date the abovementioned study by Bornstein et al. (2008) investigating *N* = 220 dyads from the United States, Argentina and Italy, is the only published study of cross-country comparisons using the EAS. In this study, the scores in *structuring* and *sensitivity* of Italian mothers were higher than the ones of mothers from the United States and Argentina. In a study by Derscheid (2012) and Derscheid et al. (2019) with subcultural comparisons in the United States, no significant differences in EA were found between African American, Caucasian and Hispanic mother-child dyads.

Several prevention or intervention studies using the EAS exist, indicating that they are sensitive to change (Biringen et al., 2014). This was reported in two studies on adopted children, one on internationally adopted children compared to non-adopted ones in the United States by Garvin et al. (2012) and one by Van den Dries et al. (2012) on children adopted mostly from China, but also some other countries by families in the Netherlands. In the study by Garvin et al. (2012), parental EA predicted post-institutionalized children’s improvement of social adjustment over time. Van den Dries et al. (2012) found children whose adoptive mothers were more *sensitive* to show less indiscriminate friendliness and former foster care children to improve to a higher extent than former institutionalized children regarding their *responsiveness* after adoptive placement. Furthermore, studies using an EA intervention training, showed an enhancement of EA. This was the case, also for adopted children, in an EA parent intervention, but not in a control group (Baker et al., 2015) and for both children (child *responsiveness*) and teachers (*structuring*) in an EA child care intervention, also compared to a control group (Biringen et al., 2012). In a recent study with a brief parenting intervention with only one session for middle class mother-child dyads from 0 to 3 years (McConnell et al., 2020), only the child dimensions showed significant improvement. In an attachment-based intervention study with multi-cultural, pregnant adolescents in Australia (Nicolson et al., 2013) the intervention group scored significantly higher than the comparison group in two subscales (maternal *non-intrusiveness* and maternal *non-hostility*) in a free play situation and additionally in maternal *sensitivity* in a free play plus separation-reunion setting. Both, in the Nurse Family Partnership study on low-income mothers and their infants (Olds et al., 2002) as well as in a study using Parenting Child Interaction Therapy on families at risk for child abuse (Thomas and Zimmer-Gembeck, 2011) parental *sensitivity* improved significantly more in the intervention compared to a control group. Recently, a Parenting Child Interaction Therapy with toddlers (Kohlhoff et al., 2020) showed, amongst other things, positive effects at a community-based Australian child behavior treatment clinic regarding all EA scales for two different time points post-treatment compared to a waiting group (directly after and at 4 months follow-up). Salomonsson and Sandells (2011a) showed in a randomized controlled trial comparing two groups of mother-infant dyads in Sweden, amongst other things, that maternal *sensitivity* improved significantly more in the mother-infant psychoanalytic treatment (MIP) plus CHCC compared to only Child Health Centre care (CHCC). They showed in an additional study, including qualitative characteristics of mothers and babies, that maternal *sensitivity* improved significantly more for an “ideal type” of babies they created and called “affected” by disturbance (in contrast to “unaffected” ones), in the MIP than in the CHCC condition (Salomonsson and Sandells, 2011b).

In a longitudinal perspective, in a four wave study of a subsample of a community based sample in Canada of mother-child dyads (child age 6–55 months) without an intervention (Matte-Gagné et al., 2018), EA among the mothers turned out to be stable, whereas child EA significantly increased during infancy and the toddler years into preschool. To our knowledge, the EAS have not been used yet, in order to evaluate the effects of a psychoanalytically oriented intervention for immigrant background and Meurs et al. (2006) did not apply the EAS or another attachment related instrument in their study (Meurs and Jullian, 2015). Thus, our study is a pilot study in this respect.

Regarding variables influencing or interacting with parental and child EA at one time point or predicting it across time, there is some evidence that maternal education, maternal perceived social support, age of the mother, age of the child and child gender may play an important role (Bornstein et al., 2012; Matte-Gagné et al., 2018; MacMillan et al., 2021). For child gender, the reported results are inconsistent, some reporting no differences (Robinson et al., 1993; Biringen et al., 1994), while others (Lovas, 2005; Bornstein et al., 2008; Matte-Gagné et al., 2018), indicate higher EA scores for mothers in interaction with daughters than with sons as Matte-Gagné et al. (2018) sum up. Child gender is generally associated with differing emotional reactions of the parents (Biringen et al., 1999; Bornstein et al., 2010). For maternal education, as an indicator of SES, there has also been found a relation to maternal EA (Bornstein et al., 2007; van Doesum et al., 2007), but as Matte-Gagné et al. (2018) point out, mostly not in a longitudinal perspective. SES in general seems to influence both parenting behavior, implicit parenting motives or health, socio-emotional and cognitive development of children (Ziv et al., 2000; Bradley and Corwyn, 2002; McCarthy et al., 2003; Chaudhuri et al., 2009; Weis and Toolis, 2010; Durgel et al., 2012; Emmen et al., 2013; Chasiotis et al., 2014). Furthermore, access to social support seems to play an important role for maternal EA during infancy both at one point of measurement (van Doesum et al., 2007; Stack et al., 2012), and in a longitudinal perspective (Matte-Gagné et al., 2018) with higher levels of social support predicting higher levels of maternal *sensitivity* and *structuring*. Matte-Gagné et al. (2018) found the same for the level of maternal education. However, when social support was controlled this relation disappeared, and additionally they found a prediction of lower levels of maternal *hostility* by level of maternal education. In addition, the age of a child is supposed to influence the complexity of mutual exchanges (Biringen, 2008) and, the age of the mother is considered to influence maternal characteristics (Kermoian and Leiderman, 1986; Berlin et al., 2002).

Considering the reported results on the EAS’ sensitivity to change due to an intervention as well as on stability especially of the parental dimensions over time without an intervention, the scales seemed to be a suitable instrument for our study in order to investigate a potential change in the emotional relationship between the participating mothers and their children due to our program. Still, in sum, the findings on variables predicting parental and child EA across time are inconsistent and further research is needed.

## Aims and Hypotheses

The project FIRST STEPS (Leuzinger-Bohleber and Lebiger-Vogel, 2016) aimed at evaluating the implementation as well as the short- and long-term effectiveness of psychoanalytically oriented, professionally supported early parenting intervention A compared to the outcomes of intervention B, led by paraprofessionals (both will be described in more detail below) in a RCT design (Lebiger-Vogel et al., 2015). Amongst others, it was expected that at time of measurement t2 (post treatment, at approximately 3–3.5 years of age) the children in intervention A show a significantly greater improvement in the quality of EA, measured by the Emotional Availability Scales (EAS, Biringen, 2008) in interaction with their mothers compared to the children in intervention B in relation to a baseline measurement (t1), when the children were approximately 5–11 months old. This is the topic of this sub-study. Since longitudinal studies suggest stable parental EA over time without an intervention during infancy and the toddler years (Matte-Gagné et al., 2018), it is expected, that if effects are found, they will mostly be attributable to intervention A or B, respectively. For children it is more of an open question, whether intervention or developmental effects will be predominant, since Matte-Gagné et al. (2018) found a change in both of the EA child dimensions over time, attributable to natural developmental processes.

The current study aimed a examining the following questions:

1. Do the mother-child dyads in intervention A show a significantly higher increase of EAS scores post treatment compared to the mother-child dyads who received intervention B?
2. Are these intervention effects only found on the maternal dimensions of the EAS or also on the child dimensions?

## Materials and Methods

### The Intervention

In this study, two prevention programs for immigrant families were compared, both lasting from pregnancy to entry into kindergarten, i.e., in Germany around the age of three: one psychoanalytically oriented, more complex and individual, the FIRST STEPS intervention (A) and a standard care intervention with paraprofessional group leaders (B) [see Lebiger-Vogel et al. (2015, 2020) for a detailed description of the two interventions]. A randomized comparison group design was pursued to explore differentiated effectiveness of FIRST STEPS in comparison to a layperson-assisted prevention offering in two major cities in Germany (Frankfurt/Main and Berlin). In Frankfurt/Main offerings took place at integration course (language courses for recently immigrated persons in Germany) providers; in Berlin at the Vivantes Clinic Neukölln in collaboration with the children’s house of health e.V, a clinic where 70% of mothers giving birth have an immigrant background.

### Intervention A – FIRST STEPS

As described before in more detail (see Lebiger-Vogel et al., 2015), the mothers^2^ were supported by psychoanalytically trained female project staff, who were mostly mothers with an immigrant background themselves, mainly in moderated weekly mother-child groups (of 6–8 mother-child dyads) conducted by two project staff members (duration: 1.5 h) and if necessary in individual contacts (*via* telephone, home visits). The mothers and children were ideally supported from the time of birth until the children entered kindergarten. The training of staff included a curriculum, psychoanalytic (case-)supervision with child and adolescent psychotherapists, and a regular reflection on group dynamics and topics with the coordinator of the practical implementation^3^. The manualized curriculum is based on empirical and psychoanalytic developmental psychology, and was developed by the Frankfurt coordinator, Claudia Burkhardt-Mußmann. Its conceptualization is based on other already evaluated psychoanalytically oriented parenting programs (Parens et al., 1995; Emde and Robinson, 2000; Meurs et al., 2006), especially on the FIRST STEPS project in Belgium, conducted by Meurs et al. (2006) [see Meurs and Jullian (2008, 2015)]. As described by Lebiger-Vogel et al. (2015) the practice staff was trained to develop a psychoanalytic “mind set” (including transference and countertransference processes) and to assume a “holding” (Winnicott, 1953) and “containing” function (Bion, 1963) during group sessions and in contact with each woman^4^. This also helped them to gain a deeper understanding of the situation of mothers in the vulnerable phase of their early motherhood as well as the needs of the children, and enabled them to serve as role models and a “secure base” (Bowlby, 1969, p. 325). The focus thus was on the individual needs of mothers and children, questions and concerns of families about the development of their child as well as questions in connection with the immigration of the families (support in contact with social and psychiatric services, language courses, institutions, clinics, educational institutions, etc.). In this way, the project staff supported parenting skills (e.g., reflective functioning, adequate emotion regulation) and coping with difficulties related to recent immigration. In a typical psychoanalytic approach, the topics of the group meetings were not fixed, rather themes, which were brought up by the participants during the meetings were discussed. Our approach as well as the Belgian one (see above) can best be described as “Relationship-based development counseling” (Lebiger-Vogel et al., 2020, p. 25, for conceptual differences see also Lebiger-Vogel et al., 2020).

### Intervention B

In intervention B (see Lebiger-Vogel et al., 2015) the mother-child groups took place with the same frequency and duration (also weekly group meetings, duration 1.5 h, 6–8 mother child dyads). Group leaders were two female paraprofessionals, also mainly mothers, all with an immigrant background (a common format in Germany, see Friedrich and Siegert, 2009). They were encouraged to pass on their experiences to the mothers and to invite for exchange, as a kind of “helping people help themselves,” but were only instructed and informed about the study very basically, including the frequency and duration of the weekly group meetings, the aimed duration of the intervention until children enter kindergarten and the research instruments. Otherwise, they were free to lead their groups according to their views and experiences as immigrants and mothers. They received no content-related support and apart from contact to the research team regarding organizational questions and collecting data had no contact with the project implementation (see Lebiger-Vogel et al., 2015).

## Design of the Study

**FIGURE 1:**
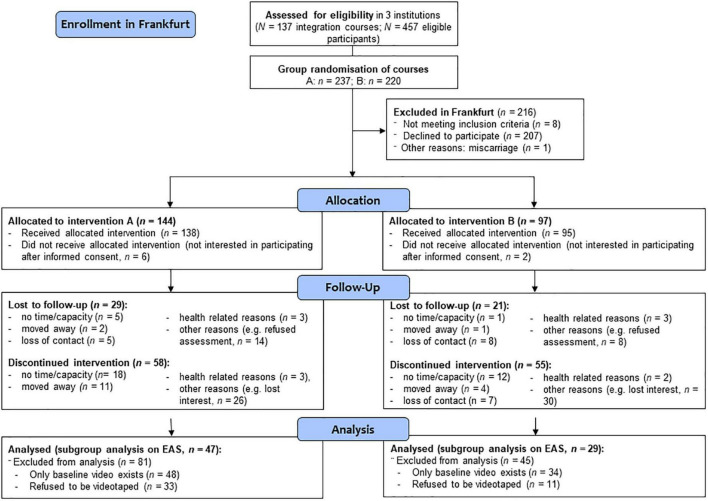
Flowchart of participants and randomization in Frankfurt/Main.

**FIGURE 2:**
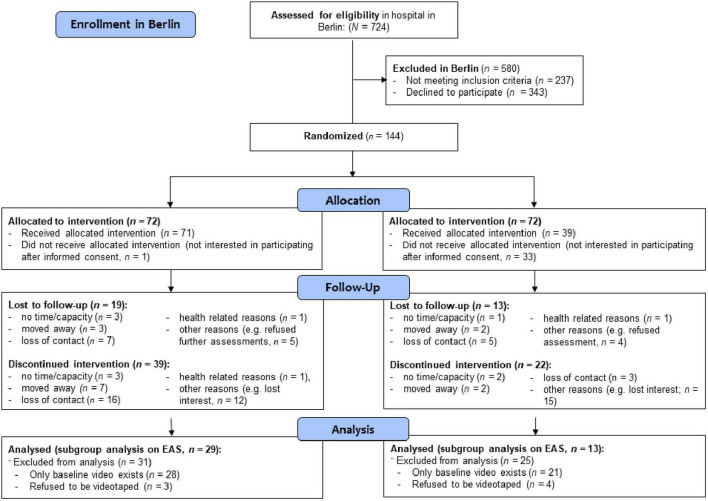
Flowchart of participants and randomization in Berlin.

The longitudinal study (3 years intervention) was carried out in a RCT design with two different recruitment strategies, as described above (for detailed information see Lebiger-Vogel et al., 2015, 2020). In Frankfurt/Main integration courses (language courses) of three collaborating institutions served as clusters and were randomized, because women from the same integration course could not be referred to different intervention offerings (cluster-randomization) (see Figure 1). In Berlin, at a maternity unit of a large hospital, the Vivantes clinic Neukölln first generation immigrant mothers, also with little knowledge of the German language, were informed about the study as soon as possible, when visiting the clinic in order to carry out a single randomization, when they agreed to participate (see Figure 2). The participants were all blinded to their group assignment, that is, if they were participating in intervention A (FIRST STEPS) or in intervention B as described in more detail before (Lebiger-Vogel et al., 2015).

As described before (see Lebiger-Vogel et al., 2015), the sample size calculation and power analysis is based on α = 0.05 at a power of 0.80. At least small effect sizes were expected, leading to a number of *N* = 72 participating mother-child dyads ideally selected per treatment.

## Participants

All participants, who agreed to be videotaped at both times of measurement, were included in this sub-study. Thus a sub-sample of mother-child dyads (*N* = 118; see Table 1) from 37 different countries who had recently moved to Germany was included in this study^5^. Informed written consent was obtained from all of the participating mothers (see Lebiger-Vogel et al., 2016). The three largest groups were the following: *n* = 34 (28.8%) of the mothers had Sub-Saharan origin, mostly coming from Ghana (8.5%), Ethiopia (6.8%) or Eritrea (5.9%), as well as from Kenya, Nigeria, Somalia and Sudan (together: 7.5%). *N* = 19 women (16.1%) originally came from North Africa, mainly from Morocco (8.5%) and Algeria (5.9%) as well as from Egypt (1.7%) and *n* = 18 mothers (15.3%) were from Turkey. The majority of the women reported to be Muslims (53.7%), followed by Christians (33.3%). The age of the mothers at the first time of measurement varied between 18.46 and 44.05 years (*M* = 31.25, *SD* = 4.94). Average age of the mothers was almost identical in both groups (A: *M* = 31.00, *SD* = 5.20; B: *M* = 31.70, *SD* = 4.46).

**TABLE 1:** Sample description of *N* = 118 mothers and children.

		Total sample	Intervention A *n* = 76	Intervention B *n* = 42
Mean age of mothers at baseline (in years)		31.25 (*SD* = 4.94)	31.00 (*SD* = 5.20)	31.70 (*SD* = 4.46)
Family status	Married/in relationship Single	78% 22%	82% 18%	76% 24%
Education	Mean duration of school education (in years)	11.10 (*SD* = 3.02)	10.85 (*SD* = 3.03)	11.69 (*SD* = 2.99)
Highest educational qualification	A-levels/highschool-diploma Lower secondary education Minimal school education No school graduation	53.7% 11.4% 12.3% 21.4%	57.9% 11.8% 11.8% 20.9%	51.5% 12.1% 15.2% 22.6%
Religious affiliation	Muslim Christian Other religion No religion	53.7% 33.3% 8.4% 2.8%	55.6% 33.3% 7.0% 4.2%	50.0% 33.3% 11.1% 0%
Mean age of children (in months)	At baseline (t1) Post treatment (t2)	9.67 (*SD* = 7.15) 41.41 (*SD* = 5.78)	9.47 (*SD* = 6.88) 40.32 (*SD* = 4.90)	11.42 (*SD* = 7.47) 43.36 (*SD* = 6.72)
Sex of children	Female Male	45% 55%	42% 58%	50% 50%
ADS (CES-D)		14.47 (*SD* = 8.34)	14.77 (*SD* = 8.43)	13.52 (*SD* = 8.00)
HBS-L (overall strains of the family)		0.68 (*SD* = 0.85)	0.83 (*SD* = 0.87)	0.49* (*SD* = 0.82)

Reported are either means or percentages, SD, standard deviation; * significant differences between the two groups (A and B) using a Mann–Whitney U-Test.

Mean level of school years was 11.10 years (*SD* = 3.02). 21.4% (*n* = 21) of the women reported no graduation, 78.6% (*n* = 77) reported having graduated either in their country of origin, in Germany or in both countries, 16.9% (*n* = 20) did not specify their level of education. Of those reporting a graduation, mostly in their country of origin, but some also in Germany, 12.3% reported a minimal level of school education, (*n* = 14), 11.4% reported lower secondary education (*n* = 13), 53.7% had A-levels/highschool-diploma (*n* = 61). Of those having a graduation, 34.3% reported a college degree (*n* = 34) and 35.6% (*n* = 42) reported to have a different kind of vocational qualification, but only 5.9% (*n* = 7) had already worked in the profession they had been qualified in in Germany.

Concerning family status, 16.1%, of the women reported to be a single parent (*n* = 19) or to be widowed, divorced or separated (5.9%, *n* = 7), altogether 22% (*n* = 26). The majority of the women were married (73.7%, *n* = 87) or lived together with their partner without being married (4.2%, *n* = 5), together 78% (*n* = 92). The age of the children at the first time of measurement varied between 1.93 and 28.03 months (*M* = 9.67, *SD* = 7.15). They were 45% female (*n* = 53) and 55% male children (*n* = 65).

## Study Procedures and Instruments

### Assessments

In order to assess the main outcome of this study the Emotional Availability Scales (EAS, Biringen, 2008, 4th edition, version for children from 0 to 5) were applied. As noted above, the EAS consist of six dimensions (4 about the caregiver and 2 about the child). Each dimension can be scored on a dimensional scale with values from 1 to 7 in a *direct global score* (and in a total score with subscales, not being reported here), a higher score indicating a higher EA. Independent raters blind rated videos from a free-play mother-child interaction, where the instruction to the mothers was to interact with their child as usual. Other studies reported partly good, partly excellent contextual retest (0.79–0.92) and interrater reliabilities (0.76 and 0.96), which seems to hold independently from setting (home or laboratory environment; see Biringen et al., 2014). After being trained and certified by the developer of the EAS, Zeynep Biringen, all four raters in the present study were blinded to the mother-child dyads intervention group (see also Rickmeyer et al., 2017). For the global ratings they achieved an interrater reliability of ICC 0.94–1.0 (average-measure intra-class correlations), which indicates excellent agreement (Cicchetti, 1994).

Observation length was scheduled 30 minutes, stemming from findings showing reliability increasing with observation length (Aviezer et al., 2003; Robinson and Emde, 2004; Bornstein et al., 2006a,b).

The following potential confounding variables (see Bornstein et al., 2012) were tested due to their relevance indicated by previous studies either descriptively or inferential statistically: age of the child, sex of the child, age of the mother, depressive symptoms, overall strains of the family (as an indicator of family resources/social support), level of education of the mothers in years (as an indicator for SES), partnership (as an additional indicator of mothers’ resources/social support). Depressive symptoms and strains of the mothers were included due to their associations with a healthy emotional exchange with their child (Carter et al., 2001; Trapolini et al., 2008; van Ee et al., 2012).

Depressive symptoms of the mothers were assessed at baseline (t1) with the German version of the Center for Epidemiological Studies-Depression Scale (CES-D; Allgemeine Depressions Skala-Langform; ADS-L; Hautzinger and Bailer, 2002), a widely used standardized self-report screening instrument with good psychometric criteria (reliabilities between 0.51 and 0.92; Hautzinger et al., 2012).

The overall strains of the family were also assessed at baseline (t1) with help of the Heidelberg Stress Scale (Heidelberger Belastungsskala, HBS-L; Stasch, 2007), a standardized screening instrument that allows to estimate family’s stresses and resources (amongst others their social support) and shows satisfying psychometric criteria (Sidor et al., 2012).

Due to the fact that not all of the mothers agreed to be videotaped, or to attrition over the course of the intervention, the comparability of the two groups A and B at baseline was not taken for granted, even though the RCT design was pursued as described. Thus, both groups were compared regarding all relevant sociodemographic variables at baseline (see section “Introduction”). In almost all of these variables no significant baseline difference between the groups A (*n* = 76) and B (*n* = 42) were found using a *t*-test, a Mann–Whitney-*U*-test, when assumptions for parametric testing were violated or for nominal variables a Chi Square test. Only in “overall strains of the family,” assessed with the HBS-L, significant differences using the Mann–Whitney-*U*-test (used due to violation of normal distribution) were found (see Table 1) and it correlated with post-treatment maternal *sensitivity*, leading to its inclusion in later analysis. Level of maternal education (in school years) was also included as a covariate for maternal *sensitivity* due to it’s potential, but especially in a longitudinal perspective still unclear influence especially on this dimension in previous studies (Biringen et al., 2014; Matte-Gagné et al., 2018). Depression scores assessed with the ADS (see Table 1) were low on average and scattering in both groups, thus this measure was not included in later analysis. Additionally, ADS scores were below the clinical cut-off score of 22 in both groups, which suggests a non-clinical sample.

### Statistical Analyses

Since the design of this study is an analysis of variance design with repeated measures (see Lebiger-Vogel et al., 2015), the methods used for evaluation were analysis of variance (ANOVA) and analysis of covariance (ANCOVA) models. The between-subjects factor is the “intervention group” (A or B) and the within-subject factor is the “time of measurement,” thus the initial value (baseline value) in each of the 6 dimensions of the EAS compared to the value in the EAS dimensions after the intervention (t2 value). SPSS Version 23.0 was used for all statistical analyses.

Additionally to ANOVAs with each of the EAS dimensions, the described characteristics were added as covariates to the models.

Since both, ANOVA and ANCOVA are relatively robust against violations of assumption of normality and of homoscedasticity (Olejnik and Algina, 1984; Tabachnick and Fidell, 2007), despite the violation of normal distribution in the included variables and homoscedasticity in some of them (baseline *sensitivity*, *responsiveness* and *involvement*, *sensitivity* at time of measurement t2), the AN(C)OVA design was still pursued.

## Results

Table 2 shows the mean values of the six different EA dimensions pre- and post-treatment. Testing the relationship between them at baseline and post treatment (at time of measurement t2) revealed a significant correlation between all of them (pre-post: mother’s *sensitivity*: *r* = 0.55, *p* = 0.00; *structuring*: *r* = 0.51, *p* = 0.00; *non-intrusiveness*: *r* = 0.39, *p* = 0.00; *non-hostility*: *r* = 0.48, *p* = 0.00; child *responsiveness*: *r* = 0.49, *p* = 0.00; child *involvement*: *r* = 0.39, *p* = 0.00). All correlations were highly significant with non-parametric tests, too.

**TABLE 2:** Mean scores of the EAS and standard deviations of *N* = 118 mother-child-dyads at baseline (t1) and at time of measurement t2 (post treatment).

EAS Scales	Mean base (*SD)*	Minimum base	Maximum base	Mean t2 (*SD)*	Minimum t2	Maximum t2
Mother’s sensitivity	4.58 (1.32)	1.00	7.00	4.57** (1.24)	1.00	7.00
Mother’s structuring	4.35 (1.49)	1.00	7.00	4.28** (1.46)	1.00	7.00
Mother’s non-intrusiveness	4.52 (1.31)	1.00	7.00	4.64** (1.27)	1.00	7.00
Mother’s non-hostility	5.61 (1.09)	2.00	7.00	5.50** (1.04)	2.00	7.00
Child responsiveness	4.45 (1.21)	1.50	7.00	4.73** (1.14)	1.50	7.00
Child involvement	3.72 (1.34)	1.00	7.00	4.65** (1.22)	2.00	7.00

** correlations p < 0.01 between time of measurement t1 and t2, SD = standard deviation; also all the EA scales at baseline and all the EA scales post treatment show highly significant correlations (p < 0.01) with each other.

However, the ANOVAS with repeated measures for each of the EAS dimensions revealed no significant main effects and no interaction effects for the parental dimensions. Only for maternal *sensitivity* the interaction effect was found to be almost significant, with a small effect size [*F*(1, 116) = 3.49, *p* = 0.064, η^2^ = 0.03].

For the child dimensions, significant effects of “time of measurement” were found for both child *responsiveness* [*F*(1, 116) = 5.01, *p* = 0.027, η^2^ = 0.04], indicating a small effect size, as well as highly significant effects for child *involvement* of the parent [*F*(1, 116) = 45.52, *p* < 0.001, η^2^ = 0.28], for the latter with a large effect size, but again, no interaction effects with the “intervention group” factor were found. These findings hint to developmental effects in children in both child dimensions (Matte-Gagné et al., 2018). No other main effects and interactions were significant in the ANOVA models.

The significant and highly significant main effects for the child dimensions were in line with significant simple main effects, which were found when looking separately at intervention A and B. For child *responsiveness* this was the case only for the intervention group A [*F*(1, 116) = 5.99, *p* = 0.016, η^2^ = 0.05] due to an increase of the mean value (4.41–4.74) in this group from baseline to post treatment.

For child *involvement* highly significant simple main effects were found for group A [*F*(1, 116) = 32.58, *p* < 0.001, η^2^ = 0.22] and for group B [*F*(1, 116) = 17.337, *p* < 0.001, η^2^ = 0.13], again due to an increase of the mean values post treatment, but for this dimension in both groups (A: 3.71–4.65 and B: 3,74–4.66).

Additionally, for *non-hostility* simple main effects showed for the comparison group B [*F*(1, 116) = 4.24, *p* = 0.042, η^2^ = 0.04] due to a decrease of the mean value (5.77–5.43) in this group from baseline to t2 (with a slight but non-significant increase in the mean value (5.53–5.55) in the intervention A condition).

Among the potential confounding variables tested inferential statistically in ANCOVAs, controlling for “overall strains of the family,” assessed with the HBS-L [*F*(1, 109) = 6.19, *p* = 0.014, η^2^ = 0.05], and “mother’s duration of school education (in years)” [*F*(1, 103) = 5.85, *p* = 0.017, η^2^ = 0.05] both led to a highly significant and for the latter to a significant interaction effect, both with a small effect size, of “intervention group” and “time of measurement” for maternal *sensitivity* in the expected direction.

**FIGURE 3:**
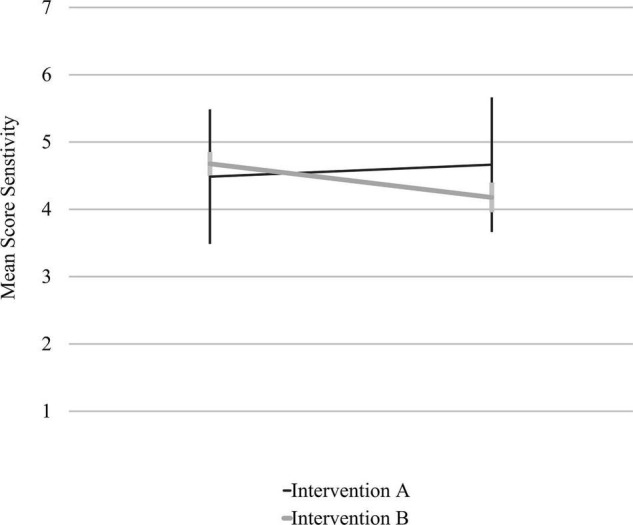
Interaction between “time of measurement” and “intervention group” for *sensitivity* in the ANCOVA model including overall strains of the family and maternal education as covariates.

Controlling for both variables in one ANCOVA led to a highly significant interaction effect of “intervention group” and “time of measurement” for maternal *sensitivity* [*F*(1, 97) = 7.98, *p* = 0.006, η^2^ = 0.08], with a medium effect size, again in the expected direction. Thus, mothers in intervention A showed a significant increase of *sensitivity* post treatment compared to the mothers who received intervention B, who showed a slight decrease (see Figure 3). No other significant interaction effects between the factors “time of measurement” and “intervention group” were found, including all other possible confounding variables for all other scales.

## Discussion

To our knowledge, this study is one of the first ones using observations of mother-child dyads from different cultural origins to shed light on variation of EA in the context of a preventive support for recently immigrated families. As far as we know, only one other study (van Ee et al., 2012) looking into EAS scores in an immigrant population in Europe exists so far. However, in the study by van Ee et al. (2012) no program was evaluated. And, as already mentioned, in the Belgian program no attachment-related outcome was assessed (Meurs and Jullian, 2008, 2015).

Our study aimed at comparing the effects of two different interventions on the relationship quality of children with an immigrant background in Germany to their recently immigrated mothers measured by the EAS. Therefore, the effectiveness of FIRST STEPS (intervention A) compared to an intervention with paraprofessional group leaders (intervention B) was evaluated in a prospective randomized comparison group design. Anticipated was that the promotion of the earliest parent-child interactions and parenting skills in the professional psychoanalytically oriented intervention (A) would have a greater positive impact on the quality of the parent-child relationship than the intervention of paraprofessionals with an immigrant background (comparison groups, B). As described, comparable intervention doses were offered in the course of the three years of intervention to ensure that effects would be caused by the type and not just by different amounts of intervention. Our hypothesis, therefore, was that all parental EA outcomes in the FIRST STEPS group would be superior. Different from what was expected, repeated AVONAs revealed no significant group differences for all four parental EAS dimensions. For the child dimensions the effect of time of measurement was highly significant, which can be interpreted as mostly natural developmental effects, a result which is in line with the findings of Matte-Gagné et al. (2018) in a longitudinal study during infancy and the toddler years. Still, on the level of simple main effects for each intervention group, only in the FIRST STEPS groups child *responsiveness* significantly improved.

In addition, when controlling for confounding variables, a highly significant medium sized interaction effect of time of measurement and group was found for maternal *sensitivity*, with an increase of the mean value in the FIRST STEPS condition, as hypothesized, and a slight decrease in the comparison group. Consistent with the findings of other studies (van Doesum et al., 2007; Stack et al., 2012; Matte-Gagné et al., 2018), controlling for overall strains of the family as an indicator of social support and for maternal education as an indicator of SES in this regard was found to be of additional explanatory value. Thus, to control for both of these confounding variables was found to be useful in detecting significant effects in favor of the FIRST STEPS condition.

This allows for the tentative conclusion that despite the non-significant results for the remaining EAS dimensions, maternal *sensitivity* as well as child *responsiveness* were influenced more positively by a participation in the FIRST STEPS intervention. It could be hypothesized that a participation in the psychoanalytically oriented professional intervention increased the mothers’ *sensitivity* toward their child’s signals and the child’s emotional response to her. The systematic group differences indicate that the broader, more individual and professional FIRST STEPS intervention, explicitly focusing on the individual needs of the participating families is more suitable to support the quality of the mother-child relationship amongst immigrant mother-child dyads than usual care. This is in line with the results of Meurs et al. (2006) regarding the efficacy of their intervention on early developmental delays in immigrant children and their school success.

It is an important question, why FIRST STEPS participation yielded effects on maternal *sensitivity* and child *responsiveness*, but not on the other EA dimensions. However, this result it is in line with the results of Salomonsson and Sandells (2011a,b) study on a psychoanalytic plus vs. only a community-based intervention. A ceiling effect might be an explanation for the absence of differential outcomes for maternal *non-hostility* as Salomonsson and Sandells (2011a) point out. Still, the simple main effect due to a decrease of *non-hostility* for the comparison group B allows for the tentative conclusion, that participation in the FIRST STEPS intervention may additionally have prevented mothers from becoming more hostile in interaction with their children when they grow older.

Nevertheless, the described effects might not be broader due to the fact, that the intervention B could, at least to some extent, also offer continuity and a “holding” experience while offering group sessions and thus could support immigrant mothers and their children in this vulnerable phase of early parenthood, too. As described, the burdens on families in the context of migration can be particularly high in the period after the birth of a child [Moro, 2014; see also Lebiger-Vogel et al. (2020)]. Support in everyday life for the young parents by experienced and trustworthy caregivers such as close relatives like grandparents and friends is often—since they are far away—not possible. Early parenthood among immigrants therefore easily leads to a situation that is overwhelming for them, accompanied by the risk of depressive withdrawal and social isolation (Leuzinger-Bohleber and Lebiger-Vogel, 2016; Lebiger-Vogel et al., 2020). All these factors as well as the described disadvantages immigrant children still have to face in Western societies stress the importance of preventive approaches with a “welcoming culture,” as was implemented and pursued in the FIRST STEPS intervention as early as possible in the lives of these vulnerable children.

## Limitations and Implications for Future Research

The generalizability of the present results is limited to immigrant mothers of the first generation in Germany. Several other limitations should be noted. First, no fathers were included in the present study, limiting its results to only one parent of the included children. As Biringen (2008) pointed out, EA is a relational construct and each dyad has its own pattern and history of EA, thus including the fathers might have, in some cases, led to different conclusions. However, we decided not to include fathers in the group meetings, since this would possibly have created quite a different atmosphere, which would have made it a lot more difficult to open up, especially for women with cultural backgrounds, where gender segregation is a common phenomenon, like in many Muslim societies. This could easily have been the case, taking into account that more than half of the participating women reported to have a Muslim religion.

Another limitation is the representativeness of the reported findings, because this study only included a subsample, which consists of those mothers who completed the 3 years intervention and not the whole original sample. However, no significant differences in baseline characteristics were found between the participants, who dropped out and the ones who finished the intervention. Additionally, both the insecure and sometimes fast changing living conditions many immigrants have to face (e.g., due to residential status issues for refugee families) as well as the long duration of 3 years contributed to the fluctuation of the participants.

In addition, it is important to take into account that natural developmental effects in the EA child dimensions, which are of course tremendous in the first years of life, seem to be predominant, leading to only slight variations due to interventions which are designed over such a long time period (see Matte-Gagné et al., 2018).

Another limitation is that due to the naturalistic design and associated practical reasons the age range of the children is quite large, both at baseline and post treatment, which is problematic regarding the interpretation of the results. However, since there are no significant differences regarding the age ranges in both groups at both times of measurement, this limitation is of minor relevance.

Furthermore, the comparability between the two institutional settings in the two cities as well as the different randomization strategies should be taken into consideration regarding the generalizability of the reported findings. However, due to this design advantages and disadvantages of a setting in two different institutional contexts in two different German major cities with a large percentage of families with an immigrant background could be explored (obligatory language courses in Frankfurt; a large maternity clinic in Berlin), providing important implication for future projects [see Fritzemeyer et al. (2019) for a detailed comparison of the two different locations].

An important methodological issue is the validation of the methods used. As described before, in at least 22 countries all over the world the EAS have been applied with validity and reliability in each of them [see review by Biringen et al. (2014)]. However, as pointed out, comparisons from a cross-cultural perspective in order to investigate possible cross-cultural differences in EA levels were only made in very few studies by now (Bornstein et al., 2008; Derscheid, 2012, see also Rickmeyer et al., 2017). The same applies for instruments assessing possible covariates and concurring predictors like depression, since its expression might vary in different cultures too (Collins et al., 2011). This tackles a commonly discussed problem—the adaptation of instruments validated in a specific sample (Geisinger, 1994) to a new language and a different cultural group, where cross-cultural validity of the applied research instruments is not guaranteed: an important subject for future studies. Factors like social desirability, gratefulness or phenomena of overcompensation in the first time after a migration (Sluzki, 2001) might have also played a role here.

However, it should be noted that the often times unstable life circumstances of the families and lacking German skills made it particularly difficult to recruit the participating families, to assess data and to conduct a longitudinal study with this sample. Implications for future research are to further shed light on which of the specific components of our program are especially useful to support immigrant families. Furthermore, it would be interesting to investigate with a larger sample differential effects of the intervention for different groups of immigrant families, e.g., those with a refugee background or women who immigrated through family migration. The findings recommend controlling for the overall strains of the family and mother’s duration of school education. In future studies it could be particularly promising to investigate how mothers’ traumatization influences the effects of the psychoanalytically based intervention on EA because studies indicate that traumatized mothers show lower EA scores. For example, in the study by van Ee et al. (2012) in the Netherlands a positive relationship between maternal posttraumatic stress symptoms and unstructuring, hostile or insensitive, but not intrusive maternal behavior was found in a sample of asylum seekers and refugees. Additionally, infants of mothers who were traumatized showed lower levels of *involvement* and *responsiveness*.

## Data Availability Statement

The raw data supporting the conclusions of this article will be made available by the authors, without undue reservation.

## Ethics Statement

The studies involving human participants were reviewed and approved by the ethic review. The Commission of the Federal Chamber of Psychotherapists of the State of Hessen, Germany, has approved the final study protocol and the final version of the written informed consent form. Written consent was obtained from each participating family. The trial has been carried out in keeping with local legal and regulatory requirements. Written informed consent to participate in this study was provided by the participants’ legal guardian/next of kin.

## Author Contributions

ML-B developed the study transcript and contributed to design and concept for intervention “First Steps.” JL-V developed the study transcript and contributed to design, assessments, statistics, and drafted this manuscript. JL-V, CR, and ML-B managed the study, supervised research staff, enrollment, and the follow-up of study participants. JL-V and CR analyzed the study data. JL-V, CR, PM, and ML-B contributed equally to writing this manuscript and approving the final version. All authors contributed to the article and approved the submitted version.

## Conflict of Interest

The authors declare that the research was conducted in the absence of any commercial or financial relationships that could be construed as a potential conflict of interest.

## Publisher’s Note

All claims expressed in this article are solely those of the authors and do not necessarily represent those of their affiliated organizations, or those of the publisher, the editors and the reviewers. Any product that may be evaluated in this article, or claim that may be made by its manufacturer, is not guaranteed or endorsed by the publisher.

## Footnotes

1 “Difficult-to-reach” refers to the difficulty to involve or reach certain groups of population in public health, social and community services.

2 The fathers were only supported occasionally in the context of individual contacts *via* home visits, phone calls etc.

3 Child and youth-psychoanalyst Claudia Burkhardt-Mußmann (in Frankfurt) and Rose Alheim (in Berlin).

4 Containment and holding refer to the project staff’s ability to contain difficult emotions of the mothers and return them to the mothers in a digestible/manageable way (for a detailed description and differentiation of both terms see Ogden, 2004).

5 This was a sub-sample of the original sample (see Lebiger-Vogel et al., 2020) consisting of the dyads completing the study with a mother-child-interaction video existing both at baseline and post treatment. The participants dropping out did not significantly differ in baseline characteristics from those completing the study.
